# Learning from biology: biomimetic carbon cells promote high-power potassium ion batteries

**DOI:** 10.1093/nsr/nwab043

**Published:** 2021-03-13

**Authors:** Wenchao Zhang, Zaiping Guo

**Affiliations:** Institute of Environmental Engineering, School of Metallurgy and Environment, Chinese National Engineering Research Center for Control and Treatment of Heavy Metal Pollution, Central South University, China; Institute for Superconducting and Electronic Materials, University of Wollongong, Australia; School of Chemical Engineering and Advanced Materials, The University of Adelaide, Australia; Institute for Superconducting and Electronic Materials, University of Wollongong, Australia

Potassium ion batteries (PIBs) are gaining traction as an alternative scalable energy storage system due to a high abundance of potassium resources (1.5 wt% in the Earth's crust), fast ion transport kinetics of K^+^ in electrolyte and low standard reduction potential of potassium (−2.93 V vs. standard electrode potential (*E*^0^)) [1–4]. The rapidly growing field of this rising technology has forced us to search for advanced electrode materials with superior electrochemical performance and scalable production methods. Building on the historical achievements of lithium and sodium ion batteries, inexpensive carbons are one of the most promising anode materials due to their good electrical conductivity, benign tailorable properties, eco-friendliness and high stability in electrolytes [5–7]. Among all the carbon-based materials, carbon nanotubes (CNTs), graphite, graphene, amorphous carbon and their derived 3D structures have all been considered as potential candidates that could be applied in a wide range of electrochemical fields [8]. Although current studies have made a few breakthroughs to prolong the cycling stability of carbon-based electrodes, the capability for fast charging/discharging still needs to be improved for practical applications. More importantly, the use of carbon-based anodes in full cells is essential to help evaluate the feasibility for this un-matured technology.

**Figure 1.**
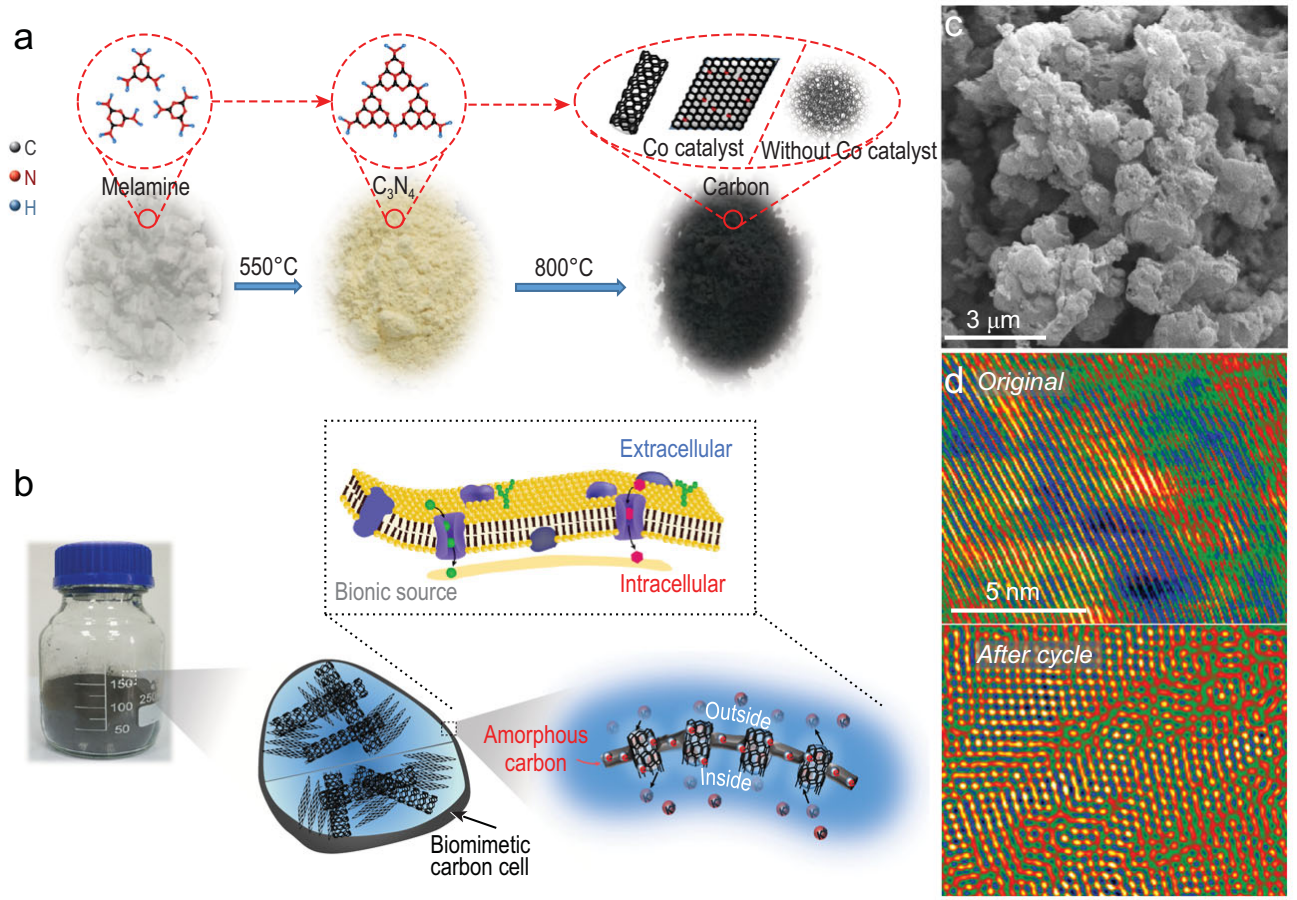
A schematic illustrating (a) the synthesis route for preparing biomimetic carbon cells (BCCs), and (b) the structural and functional similarity of membranes in a biological cell and a BCC; (c) scanning electron microscopic (SEM) image of the BCCs; (d) transmission electron microscopic (TEM) images of graphite present in the BCC before and after 1000 cycles [10].

Inspired by biological cells demonstrating natural selection over billions of years, it has been found that metal ions selectively absorb and accumulate within the cells of halophytic plants, which then can be converted into graded 3D carbon and metal oxide nanocomposites [9]. Based on this interesting finding, Lu and his co-workers have prepared biomimetic carbon cells (BCCs) akin to the biological cells with several ion transportation channels preserved [10]. The synthesis of BCCs is schematically shown in Fig. 1a. It is noted that the addition of metal Co catalysts into C_3_N_4_ intermediate prepared by heating melamine precursor could promote the growth of carbon nanotubes inside the BCC. Also, the amorphous carbon that grew on the nanotubes and graphene could act as the shell to protect the entire BCC. Specifically, Fig. 1b exhibits some internal spaces inside the biological cell with surfaces composed of bilayer lipid membranes. The interior of a BCC consists of open spaces that could allow ions to transfer quickly inside the composite, thus benefiting the rate capability. In addition, the internal space of the BCC could also accommodate volume variations upon cycling, which can maintain the structural integrity of the carbon material. Figure 1c shows the morphology of the BCCs, which exhibit a characteristic ellipsoidal shape similar to that of biological cells. Interestingly, the graphitic structure inside the BCC could still be maintained after cycling for 1000 cycles, as evidenced by the lattice fringe images shown in Fig. 1d. In terms of electrochemical performance, half-cell tests suggested that the BCC anode could operate over 15 months at a current density of 100 mA g^–1^. Impressively, the authors have also evaluated the full-cell performance (Prussian Blue as cathodes and BCC as anodes). This full cell shows a practical voltage range of discharge/charge curves and ultra-stable cycle performance at a current density of 500 mA g^–1^.

The biological fabrication approach presented in this study holds great promise for electrode material synthesis for future low-cost and high-power PIBs. More importantly, this biomimetic strategy is promising for the construction of 3D multifunctional materials. This study will be of broad interest to scientists involved in materials science and energy storage. This BCC material might be further optimized via more uniform distribution of CNTs in the BCC 3D architecture. For instance, the CNTs could homogeneously grow inside BCCs via controlling the size and distribution of Co catalysts. Further research on carbon-based electrode materials for PIBs could focus on (i) the increase of graphitic carbon inside the composite electrodes to enhance the energy density; (ii) the introduction of heteroatom-doping to increase active sites and facilitate ion transportation; and (iii) the optimization of electrolyte to form stable and robust solid electrolyte interphase layers to reduce unwanted side reactions.

***Conflict of interest statement*.** None declared.
